# Omega-3 fatty acid supplementation as an adjunctive therapy in the treatment of chronic kidney disease: a meta-analysis

**DOI:** 10.6061/clinics/2017(01)10

**Published:** 2017-01

**Authors:** Jing Hu, Zuoliang Liu, Hao Zhang

**Affiliations:** I The Third Xiangya Hospital of Central South University, Department of Nephrology, Changsha 410013, China.; II The Third Xiangya Hospital of Central South University, Medical Intensive Care Unit, Changsha 410013, China.

**Keywords:** Omega-3 Fatty Acid, Adjunctive Therapy, Chronic Kidney Disease, Meta-analysis

## Abstract

The aim of this study was to evaluate the benefits and risks of omega-3 fatty acid supplementation in patients with chronic kidney disease. A systematic search of articles in PubMed, Embase, the Cochrane Library, and reference lists was performed to find relevant literature. All eligible studies assessed proteinuria, the serum creatinine clearance rate, the estimated glomerular filtration rate, or the occurrence of end-stage renal disease. Standard mean differences with 95% confidence intervals for continuous data were used to estimate the effects of omega-3 fatty acid supplementation on renal function, as reflected by the serum creatinine clearance rate, proteinuria, the estimated glomerular filtration rate, and relative risk. Additionally, a random-effects model was used to estimate the effect of omega-3 fatty acid supplementation on the risk of end-stage renal disease. Nine randomized controlled trials evaluating 444 patients with chronic kidney disease were included in the study. The follow-up duration ranged from 2 to 76.8 months. Compared with no or low-dose omega-3 fatty acid supplementation, any or high-dose omega-3 fatty acid supplementation, respectively, was associated with a lower risk of proteinuria (SMD: -0.31; 95% CI: -0.53 to -0.10; *p*=0.004) but had little or no effect on the serum creatinine clearance rate (SMD: 0.22; 95% CI: -0.40 to 0.84; *p*=0.482) or the estimated glomerular filtration rate (SMD: 0.14; 95% CI: -0.13 to 0.42; *p*=0.296). However, this supplementation was associated with a reduced risk of end-stage renal disease (RR: 0.49; 95% CI: 0.24 to 0.99; *p*=0.047). In sum, omega-3 fatty acid supplementation is associated with a significantly reduced risk of end-stage renal disease and delays the progression of this disease.

## INTRODUCTION

People with chronic kidney disease are at a particularly high risk of developing end-stage renal disease (ESRD) and all-cause mortality 1,2. The addition of omega-3 fatty acid supplements to the diet has been well documented to affect the risk factors for cardiovascular diseases 3,4. Over the past few decades, many studies have shown that omega-3 fatty acids are also clearly effective at reducing proteinuria in patients with chronic glomerular disease 5,6. In addition, the effect of omega-3 fatty acids on proteinuria is dose dependent and associated with a dose-dependent effect on plasma phospholipid eicosapentaenoic acid (EPA) and docosahexaenoic acid (DHA) levels. These facts are worth exploring further 7. However, the effects of omega-3 fatty acids on renal function and subsequent ESRD events have not been confirmed.

Several studies have indicated that short- or long-term intervention with omega-3 fatty acids might reduce the risk of ESRD and proteinuria and increase the creatinine clearance rate (CCR) 8,9. In contrast, several other studies have shown no significant association of omega-3 fatty acids with a reduced risk of renal failure 10-16. These varying results prompted the present study, which examined the available relevant randomized controlled trials (RCTs) to determine the effect of omega-3 fatty acids on the risk of proteinuria and on the CCR, the estimated glomerular filtration rate (eGFR), and the occurrence of ESRD in patients with chronic kidney disease.

## MATERIALS AND METHODS

### Data sources, search strategy, and selection criteria

This review was conducted and reported according to the Preferred Reporting Items for Systematic Reviews and Meta-Analysis Statement issued in 2009 (Checklist S1) 17.

Any RCTs that examined the effect of omega-3 fatty acid supplementation in patients with chronic kidney disease were eligible for inclusion in the study, and no restrictions were placed on language or publication status (i.e., published, in press, or in progress). The PubMed, Embase, and Cochrane Library electronic databases were searched for articles published through October 2014, with search terms including “omega-3 fatty acids” OR “fish oil” OR “polyunsaturated fatty acids” OR “eicosapentaenoic acid” OR “EPA” OR “docosahexaenoic acids” OR “DHA” AND (“nephropathy” OR “kidney disease”) AND “human” AND “clinical trial”. Manual searches of the reference lists from all relevant original and review articles were also conducted to identify additional eligible studies. The medical subject headings, methods, patient populations, designs, interventions, and outcome variables of these studies were used to identify the relevant articles.

The literature search was independently undertaken by 2 authors using a standardized approach. Any inconsistencies between these 2 authors were reviewed by the primary author to reach a consensus. A study was eligible for inclusion if the following criteria were met: 1 design as an RCT or quasi-RCT; 2 reporting of at least 1 outcome related to proteinuria, the CCR, the eGFR, or the occurrence of ESRD; and 3 comparison of the effects of adding any dose or a high dose of omega-3 fatty acids with no or low-dose omega-3 fatty acid supplementation, respectively.

### Data collection and quality assessment

All data from eligible trials were independently abstracted, in duplicate, by 2 independent investigators using a standard protocol and were reviewed by a third investigator. Any discrepancies were resolved by group discussion, and the primary author made the final decision. The recorded variables were as follows: first author’s name, publication year, study design, type of blinding, number of subjects, mean age, percentage male, current disease status, intervention regimens, duration of the follow-up period, and outcomes of each group. Study quality was assessed using the Jadad score 18, which is based on the following 5 subscales: randomization (1 or 0), concealment of the treatment allocation (1 or 0), blinding (1 or 0), completeness of the follow-up (1 or 0), and the use of intention-to-treat analysis (1 or 0). A “score system” (ranging from 1 to 5) was also developed for quality assessment. A study given a score of 4 or higher was considered to be one of high quality.

### Statistical analyses

The results of each RCT were recorded as dichotomous frequency or continuous data. Individual study relative risks (RRs) and 95% confidence intervals (CIs) or standard mean differences (SMDs) were also abstracted from each trial before data pooling. Both fixed-effects and random-effects models were used to assess the pooled RR or SMD for any dose or a high dose of omega-3 fatty acids compared with no or low-dose omega-3 fatty acid supplementation, respectively. The results from the random-effects model presented here assume that the true underlying effect varied among the included trials 19,20. Heterogeneity of the treatment effects among studies was investigated using the Q statistic, and *p*<0.10 was considered to indicate significant heterogeneity 21,22. Subgroup analyses were also conducted for proteinuria, the CCR, and the eGFR on the basis of country, mean age, percentage male, current disease status, duration of the follow-up period, and study quality, whereas these analyses were not performed for ESRD outcome because of the small number of trials with available data. Additionally, a sensitivity analysis was performed by removing each trial from the meta-analysis 23. Here, *p* values for heterogeneity between subgroups were assessed using the Chi-square test and meta-regression 24. Several methods were also used to check for potential publication bias: visual inspection of funnel plots for proteinuria, the CCR, and the eGFR were conducted, and the Egger 25 and Begg 26 tests were used to statistically and quantitatively assess publication bias for these three parameters. All reported *p* values are two sided, and *p*<0.05 was considered statistically significant for all included studies. The statistical analyses were performed using Stata version 10.0 (StataCorp LP, College Station, TX, USA). Because of the lack of relevant data, funnel plots and the Egger 25 and Begg 26 tests were not used to analyze ESRD.

## RESULTS

**Figure 1:**
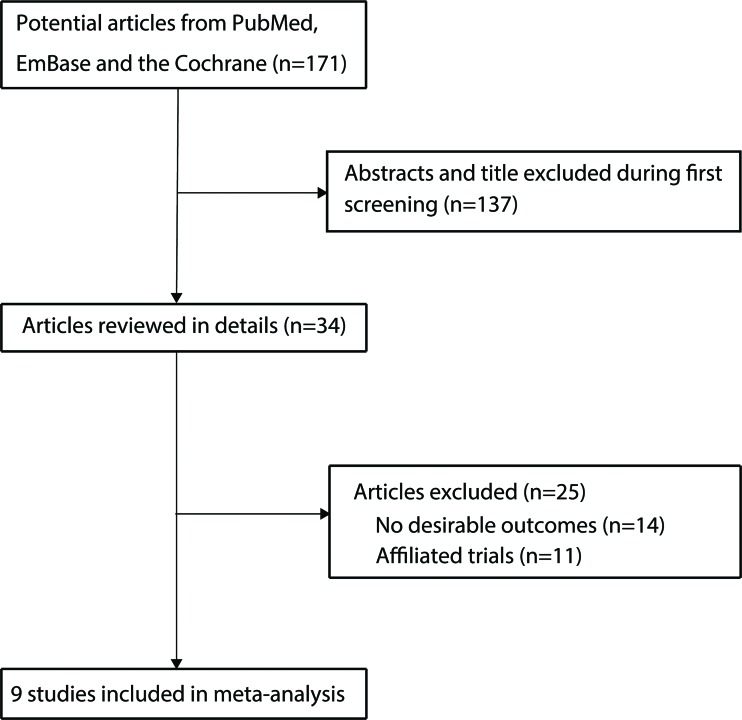
Selection of studies for the meta-analysis.

The study selection process is shown in Figure 1. We identified 171 articles in our initial electronic search, 137 of which were excluded after duplicates and irrelevant studies were removed. Therefore, 34 potentially eligible studies were queried. After detailed evaluation, 9 RCTs 8-16 were selected for the final meta-analysis. Omega-3 fatty acid supplementation for the treatment of patients with chronic kidney disease compared with no omega-3 fatty acid supplementation was studied in 8 trials 8-11,13-16; the remaining trial 12 studied high-dose compared with low-dose omega-3 fatty acid supplementation. A manual search of the reference lists of these studies did not yield any new eligible studies. The general characteristics of the included studies are presented in Table 1.

Of the 9 included trials, comprising 444 patients, with 23 to 106 per trial, the follow-up period for the participants was 2.0 to 76.8 months. Two studies 8,12 were conducted in the United States; 2 10,15, in Japan; 3 9,11,14, in Europe; and 2 13,16, in Australia. Seven studies 8-14 investigated the effect of omega-3 fatty acids on IgA nephropathy, and 2 trials 15,16 evaluated the effect on patients with chronic kidney disease. Study quality was assessed using the Jadad score 18 (Table 1), and a study with a score ≥4 was considered to be of high quality. Three studies 12,13,16 had a score of 4, while 5 studies 8,9,11,14,15 had a score of 3, and 1 study 10 had a score of 2.

**Figure 2:**
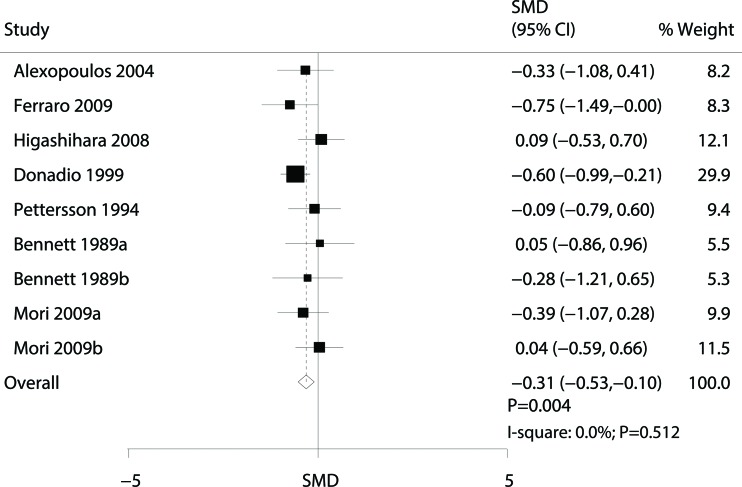
Forest plot of the association of omega-3 fatty acid supplementation with proteinuria.

Data for the effect of omega-3 fatty acid supplementation on proteinuria were available from 7 trials 8,9,11,13-16. Overall, omega-3 fatty acid supplementation was associated with a lower risk of proteinuria (SMD: -0.31; 95% CI: -0.53 to -0.10; *p*=0.004), and there was no observed heterogeneity across the trials (*p*=0.512, Figure 2).

**Figure 3:**
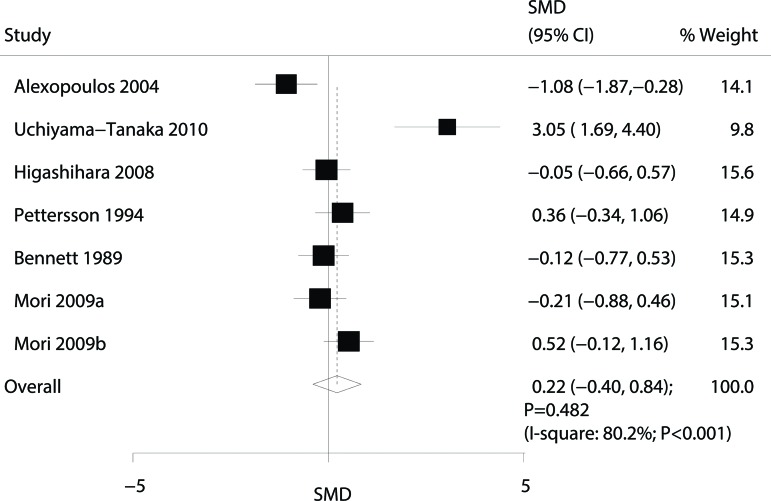
Forest plot of the association of omega-3 fatty acid supplementation with the creatinine clearance rate (CCR).

Data for the effect of omega-3 fatty acid supplementation on the CCR were available from 6 trials 10,11,13-16. Overall, omega-3 fatty acid supplementation did not have a statistically significant effect on the CCR (SMD: 0.22; 95% CI: -0.40 to 0.84; *p*=0.482). Substantial heterogeneity was detected across the included trials (*p*<0.001, Figure 3); however, based on sequential exclusion of each trial from all pooled analyses, the conclusion was not affected by the exclusion of any specific trial.

**Figure 4:**
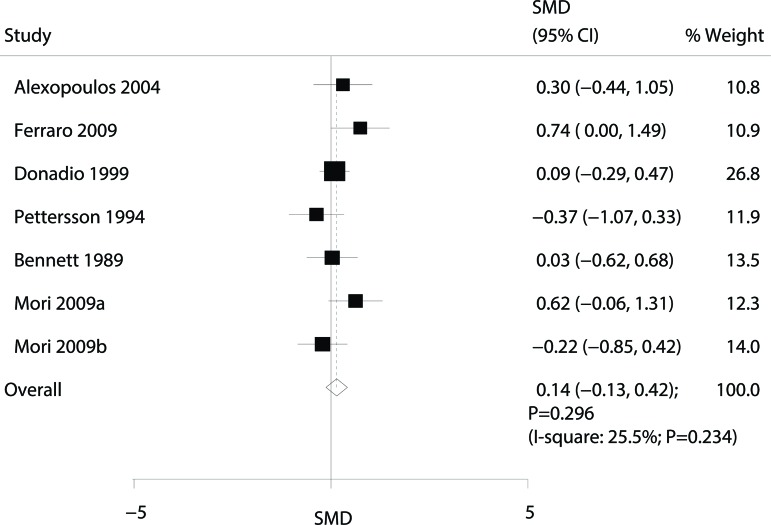
Forest plot of the association of omega-3 fatty acid supplementation with the estimated glomerular filtration rate (eGFR).

Data for the effect of omega-3 fatty acid supplementation on the eGFR were available from 6 trials 8,9,11,13,14,16. Overall, the effect of omega-3 fatty acid supplementation on the eGFR was not statistically significant (SMD: 0.14; 95% CI: -0.13 to 0.42; *p*=0.296; with nonsignificant heterogeneity; Figure 4).

**Figure 5:**
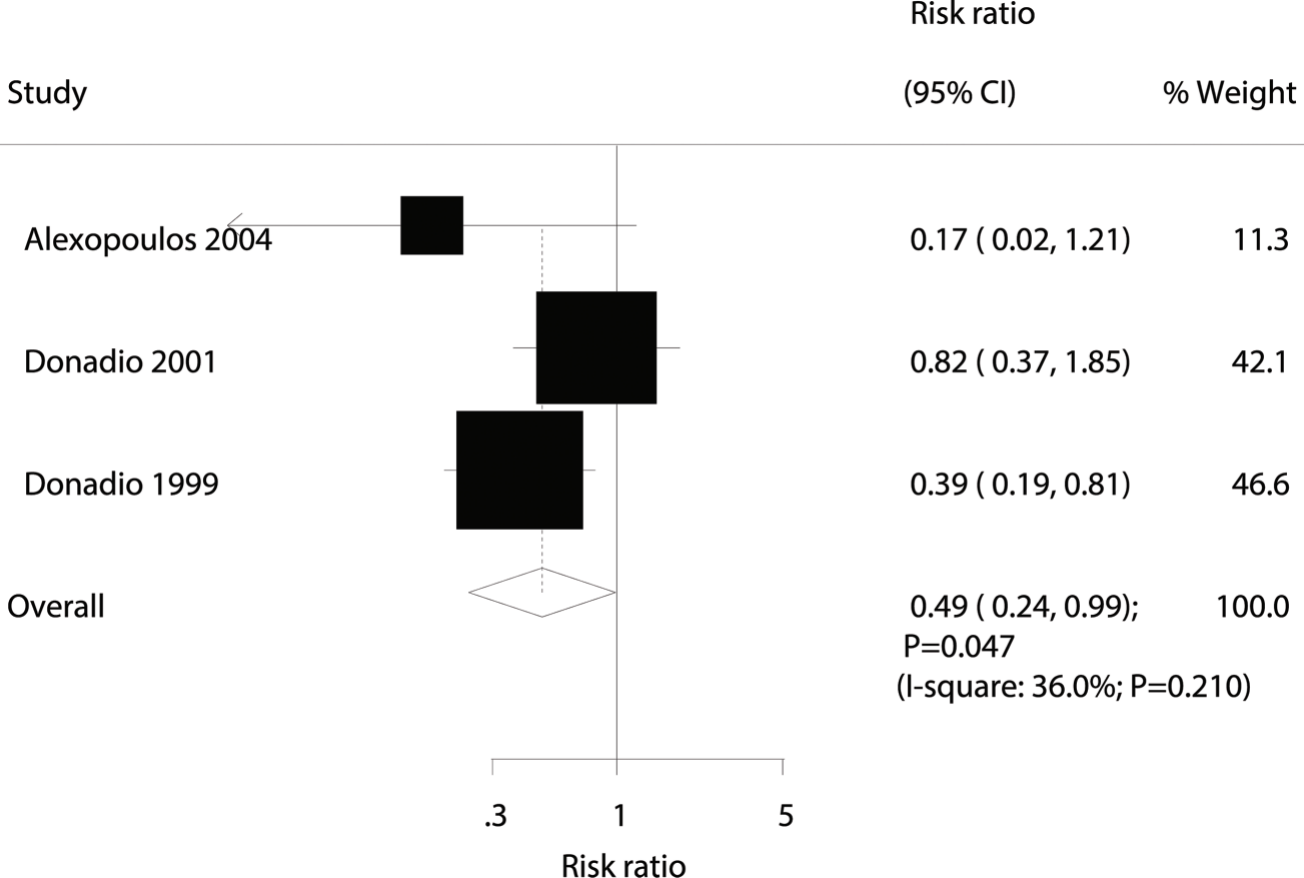
Forest plot of the association of omega-3 fatty acid supplementation with the occurrence of end-stage renal disease (ESRD).

The effect of omega-3 fatty acid supplementation on the occurrence of ESRD was examined using data from 207 patients and 44 ESRD events 8,11,12. Omega-3 fatty acid supplementation significantly reduced the risk of ESRD (RR: 0.49; 95% CI: 0.24 to 0.99; *p*=0.047; with moderate heterogeneity; Figure 5).

Subgroup analyses were conducted for proteinuria, the CCR, and the eGFR to minimize heterogeneity among the included trials and to evaluate the effect of omega-3 fatty acid supplementation on specific populations (Table 2). Omega-3 fatty acid supplementation was found to be associated with a reduction in proteinuria if the study was conducted in the United States or Australia, if the mean age was <40 years, if the percentage male was >60%, if the patients had IgA nephropathy, if the duration of the follow-up period was >24 months, or if the study was of high quality. However, there were no significant differences identified, based on predefined factors, between the effect of omega-3 fatty acid supplementation and control of the CCR and eGFR. Furthermore, subgroup analyses showed heterogeneity between subgroups for the CCR based on the follow-up duration.

Review of the funnel plots could not rule out potential publication bias for proteinuria or the CCR. In addition, the Egger and Begg tests revealed no evidence of publication bias in any case for proteinuria, the CCR, or the eGFR.

## DISCUSSION

This study was based on RCTs and explored all possible correlations between omega-3 fatty acid supplementation and proteinuria, the CCR, the eGFR, and the occurrence ESRD. This quantitative study comprised 444 individuals from 9 RCTs 8-16, with a broad range of patient characteristics. The findings from this meta-analysis suggest that omega-3 fatty acid supplementation is associated with a lower risk of proteinuria but has no significant effect on the CCR or eGFR, although it does significantly reduce the risk of ESRD. Finally, subgroup analyses indicated that omega-3 fatty acids have a significant effect on lowering the risk of proteinuria in several subpopulations.

A previous meta-analysis 27 suggested that there are insufficient data to confirm the efficacy of omega-3 fatty acid treatments for proteinuria and reduced renal function in IgA nephropathy. However, another meta-analysis 28 suggested that while omega-3 fatty acids have no benefit in terms of preserving renal function, they can ameliorate proteinuria in IgA nephropathy. In addition, the effects of omega-3 fatty acids on proteinuria were not found to be dose dependent, which was inconsistent with a previous study. The inherent limitation of the previous studies is that few included or acquired broad CIs (i.e., there was no statistically significant difference); therefore, an updated meta-analysis of RCTs was conducted to evaluate the efficacy of omega-3 fatty acids in the treatment of chronic kidney disease.

Most of the findings in the present study were in agreement with a large RCT recently published by Donadio et al. 8. This trial comprised 106 patients with IgA nephropathy who consumed 1.9 g/d EPA plus 1.4 g/d DHA and reported longer renal progression for high-risk patients with IgA nephropathy. The current study also indicated that omega-3 fatty acids significantly reduce the risk of ESRD and are associated with a lower risk of proteinuria; this may be because omega-3 fatty acids might be involved in blood pressure control, as hypertension is a strong risk factor for the progression of renal disease in patients with chronic nephropathy. In addition, the stage of the disease at the time of entry to the study might play an important role in its progression. Furthermore, there were no significant differences in the CCR or eGFR between omega-3 fatty acid-supplemented groups and control groups in this study, whereas omega-3 fatty acid supplementation was associated with a lower risk of ESRD. The reason for this finding could be that several studies reported CCR and eGFR data, whereas ESRD findings were not available, resulting in variable outcomes.

Subgroup analyses suggested that omega-3 fatty acids have a significant effect on the risk of proteinuria in several subpopulations. However, these conclusions might be unreliable because smaller trials were included in the subsets. Hence, a relative result is given, and a synthetic and comprehensive review is provided.

Three strengths of this study should be highlighted. First, only RCTs, which should have no bias compared with observational studies, were included. Second, the large sample size allowed for quantitative assessment of the association between omega-3 fatty acids and the risk of decreased renal function and proteinuria; thus, the findings are potentially more robust than those of any individual study. Third, the subgroup analyses allowed the effect of omega-3 fatty acids to be evaluated in specific subpopulations.

The limitations of this study were as follows: 1 in a meta-analysis of published studies, publication bias is an inevitable problem; 2 subgroup analyses and publication bias analyses were not performed for ESRD because of a smaller number of relevant trials, resulting in less available data; and 3 the analysis used pooled data (individual data were not available), which restricted us from performing a more detailed analysis and obtaining more comprehensive results.

The results of this study suggest that omega-3 fatty acids significantly reduce the risk of ESRD and are associated with a lower risk of proteinuria. Future trials should focus on the specific disease status to analyze the efficacy of omega-3 fatty acids in the treatment of chronic nephropathy of differing severities.

## AUTHOR CONTRIBUTIONS

Hu J, Liu Z and Zhang H contributed to the study conception and design and the data acquisition, analysis and interpretation. Hu J and Zhang H were involved in drafting the manuscript and in critically revising it for important intellectual content. All of the authors approved the final version of the manuscript to be published.

## Figures and Tables

**Table 1:** Design and characteristics of the trials included in the meta-analysis.

Source	Publication year	Country	No. of patients	Mean age, y	Percentage male (%)	Disease status	Treatment	Control	Follow-up (months)	Jadad score
Uchiyama-Tanaka and Mori (10)	2010	Japan	23	30.6	39.1	IgA nephropathy	EPA 1.8 g/d plus angiotensin-converting enzyme inhibitor (ACEI)/angiotensin-receptor blocker (ARB)	ACEi/ARB	12	2
Alexopoulos et al. (11)	2004	Greece	28	40.0	78.6	IgA nephropathy	EPA and PHA 5 g/d	Supportive treatment	48	3
Donadio et al. (12)	2001	USA	73	45.5	52.1	IgA nephropathy	2.94 g/d EPA and DHA	1.47 g/d EPA and DHA	24	4
Donadio et al. (8)	1999	USA	106	37.0	73.6	IgA nephropathy	EPA 1.9 g/d plus DHA 1.4 g/d	Placebo	76.8	4
Ferraro et al. (9)	2009	Italy	30	40.5	60.0	IgA nephropathy	RASB and PUFAs 3.0 g/dRASB	RASB	6	3
Bennett et al. (13)	1989	Australia	37	39.0	57.0	IgA nephropathy	EPA 10 g/d	Untreated	24	4
Pettersson et al. (14)	1994	Sweden	32	41.0	78.0	IgA nephropathy	Fish oil 6.0 g/d	Corn oil 6.0 g/d	6	3
Higashihara et al. (15)	2008	Japan	41	47.1	70.7	ADPKD	EPA 2.4 g/d	Untreated	24	3
Mori et al. (16)	2009	Australia	74	56.5	73.0	CKD	Omega-3 capsules 4 g/d	Olive oil 4.0 g/d	2	4

**Table 2:** Subgroup analyses of the standard mean differences (SMDs) for proteinuria and the creatinine clearance rate (CCR) following any or high-dose omega-3 fatty acid supplementation compared with no or low-dose omega-3 fatty acid supplementation, respectively.

Outcomes	Subgroup	SMD and 95% CI	*p* value	Heterogeneity (%)	*p* value for heterogeneity	*p* value for heterogeneity between subgroups
Proteinuria	**Country**					
	USA or Australia	-0.36 (-0.64 to -0.09)	0.008	0.0	0.439	0.395
	Europe	-0.38 (-0.80 to 0.04)	0.077	0.0	0.448	
	Asia	0.09 (-0.53 to 0.70)	0.784	-	-	
	**Mean age (years)**					
	40 or more	-0.21 (-0.48 to 0.07)	0.144	0.0	0.551	0.233
	<40	-0.47 (-0.80 to -0.14)	0.006	0.0	0.402	
	**Percentage male**					
	60 or more	-0.33 (-0.57 to -0.09)	0.007	8.8	0.362	0.520
	<60	-0.11 (-0.76 to 0.54)	0.736	0.0	0.624	
	**Current disease status**					
	IgA nephropathy	-0.43 (-0.70 to -0.17)	0.001	0.0	0.617	0.118
	Other	-0.07 (-0.44 to 0.29)	0.693	0.0	0.538	
	**Duration of the follow-up period (months)**					
	24 or more	-0.33 (-0.62 to -0.04)	0.026	6.1	0.372	0.749
	<24	-0.27 (-0.61 to 0.07)	0.120	0.0	0.412	
	**Study quality (Jadad score)**					
	4 or 5	-0.36 (-0.64 to -0.09)	0.008	0.0	0.439	0.548
	<4	-0.23 (-0.58 to 0.12)	0.197	3.4	0.376	
CCR	**Country**					
	USA or Australia	0.07 (-0.39 to 0.53)	0.767	31.9	0.230	0.164
	Europe	-0.34 (-1.76 to 1.07)	0.633	85.9	0.008	
	Asia	1.44 (-1.59 to 4.47)	0.352	94.0	<0.001	
	**Mean age (years)**					
	40 or more	-0.06 (-0.57 to 0.44)	0.806	63.6	0.027	0.141
	<40	1.40 (-1.70 to 4.51)	0.375	94.2	<0.001	
	**Percentage male**					
	60 or more	-0.06 (-0.57 to 0.44)	0.806	63.6	0.027	0.141
	<60	1.40 (-1.70 to 4.51)	0.375	94.2	<0.001	
	**Current disease status**					
	IgA nephropathy	0.44 (-0.80 to 1.67)	0.488	89.1	<0.001	0.918
	Other	0.09 (-0.34 to 0.52)	0.680	26.6	0.256	
	**Duration of the follow-up period (months)**					
	24 or more	-0.37 (-0.97 to 0.23)	0.224	56.5	0.100	0.005
	<24	0.76 (-0.19 to 1.72)	0.116	83.3	<0.001	
	**Study quality (Jadad score)**					
	4 or 5	0.07 (-0.39 to 0.53)	0.767	31.9	0.230	0.962
	<4	0.45 (-0.76 to 1.67)	0.464	89.0	<0.001	
eGFR	**Country**					
	USA or Australia	0.11 (-0.17 to 0.39)	0.451	7.4	0.356	0.708
	Europe	0.22 (-0.43 to 0.86)	0.510	57.2	0.097	
	**Mean age (years)**					
	40 or more	0.20 (-0.23 to 0.64)	0.363	48.7	0.099	0.631
	<40	0.08 (-0.25 to 0.40)	0.648	0.0	0.871	
	**Percentage male**					
	60 or more	0.17 (-0.15 to 0.49)	0.304	37.0	0.160	0.735
	<60	0.03 (-0.62 to 0.68)	0.927	-	-	
	**Current disease status**					
	IgA nephropathy	0.13 (-0.17 to 0.43)	0.391	18.7	0.296	0.869
	Other	0.19 (-0.63 to 1.01)	0.646	67.8	0.078	
	**Duration of the follow-up period (months)**					
	24 or more	0.11 (-0.19 to 0.41)	0.460	0.0	0.851	0.831
	<24	0.18 (-0.37 to 0.73)	0.515	61.0	0.053	
	**Study quality (Jadad score)**					
	4 or 5	0.11 (-0.17 to 0.39)	0.451	7.4	0.356	0.708
	<4	0.22 (-0.43 to 0.86)	0.510	57.2	0.097	
